# The Outcomes of Pediatric Hematopoietic Stem Cell Transplantation Recipients Requiring Intensive Care Unit Admission- A Single Center Experience

**Published:** 2019-06-11

**Authors:** Royce Kwon, Sophia Koutsogiannaki, Steven J. Staffa, Koichi Yuki

**Affiliations:** 1 Department of Anesthesiology, Critical Care and Pain Medicine, Cardiac Anesthesia Division, Boston Children’s Hospital, Boston, Massachusetts, USA; 2 Tufts University Faculty of Medicine, Boston, Massachusetts, USA; 3 Department of Anaesthesia, Harvard Medical School, Boston, Massachusetts, USA

## Abstract

**Background:**

Although the outcome of pediatric hematopoietic stem cell transplantation (HSCT) has significantly improved, it remains to be associated with high mortality. Identifying patients at high risk of mortality may potentially help to triage clinical management. The primary objective of this study is to evaluate risk factors associated with mortality of patients who received HSCT and admitted to ICU using pediatric sequential organ failure assessment (pSOFA), one of pediatric severity scoring systems in intensive care unit (ICU).

**Methods:**

We performed retrospective review of electronic medical records of pediatric patients who received HSCT and were admitted to ICU in our institution between January 2010 and June 2018. Incidence of mortality was obtained, and risk factors associated with the mortality were examined using univariate and multivariable analyses.

**Results:**

The mortality rate of pediatric HSCT patients who were admitted to ICU as a whole was 27.9%. Patients were divided into three groups based on the number of HSCT required and timing of ICU admission. Patients who received first HSCT and admitted to ICU during the same hospital stay were the majority of the study population (Group A). d(pSOFA), which was defined as the difference between maximum pSOFA and admission pSOFA, greater than and equal to 7 best predicted mortality of Group A (the area under the ROC curve 0.850; 95% CI: 0.733–0.966). Univariate and multivariable analyses showed that an increase in neurologic and cardiovascular sub scores were independently associated with higher mortality (odds ratio (OR) 2.27; 95% CI: 1.32–3.93, and OR 2.69; 95% CI: 1.21–5.99, respectively).

**Discussion:**

In our single center study, pediatric HSCT patients who were admitted to ICU demonstrated a high mortality. Risk factor analysis demonstrated that patients with the progression of neurologic and cardiovascular injuries probed by pSOFA scoring system during their ICU stay were strongly associated with mortality.

## Introduction

Hematopoietic stem cell transplantation (HSCT) is instituted to treat a wide range of diseases including lymphoma, leukemia, immune-deficiency illness and myeloproliferative syndrome in pediatric population [1]. HSCT consists of intensive myeloablative chemoradio therapy followed by stem cell rescues. Stem cell rescues are accomplished with either autologous HSCT or allogeneic HSCT. With the improvement of conditioning regimens, human leukocyte antigen (HLA) typing, prevention and treatment of serious infections, transplantation outcome has improved significantly [1], and now its survival rate exceeds 80% [2,3]. However, there is still anample room to improvetheoutcome. Knowing that the majority of patients who suffer from significant complications after HSCT are admitted to the intensive care unit (ICU) for treatment, it is critical to understand the characteristics of those patients and identify risk factors associated with poor outcomes among them.

A number of severity scoring systems have been established for patients who are admitted to ICU. They are largely divided into two systems [4]. One is the system based on the data on the first day of ICU admission. This includes acute physiology and chronic health evaluation (APACHE) scoring system, simplified acute physiology score (SAPS) and mortality prediction model (MPM). Another system is repetitive scoring system, which collects data sequentially throughout the duration of ICU stay or over the first few days, including sequential organ failure assessment (SOFA) and multiple organ dysfunction score (MODS). SOFA is a scoring system that assesses the performance of six organ systems in the body (neurologic, cardiovascular, respiratory, hepatic, renal and hematological systems) and assigns a score to each system (eachorgan score ranges from 0 to 4, total 0–24). SOFA has been shown to be a powerful tool to predict mortality from hematologic malignancies [5]. Originally created for adults, pediatric SOFA version (pSOFA) incorporating age-adjustedscoring system of cardiovascular and renal systems was proposed [6]. Matics et al. validated the pSOFA scoring system in the sepsis cohort. Using the pSOFA system, we examined risk factors of mortality of HSCT patients who were admitted to ICU in our institution.

## Methods

### Data Collection

After the Institutional Review Board (IRB) approval, data were retrospectively collected from the electronic medical record of pediatric patients (less than 18 years old) who were admitted to ICU with the diagnosis of HSCT from January 2010 to June 2018. Consent was waived by the IRB. We excluded patients who received the last HSCT before January 2010 or after June 2018, or who did not require admission to ICU following HSCT. We found 199 patientsby initial search. Among them, 104 patients met the inclusion criteria. Patients were divided into three groups (A, B, and C) as follows; Group A- pediatric patients who were admitted to ICU after their first HSCT (75 patients). Group B- pediatric patients who received HSCT were discharged home but were readmitted to ICU due to post-transplant complications (15 patients). Group C- pediatric patients who had failed HSCT(s) were admitted to ICU after an additional HSCT (14 patients). The pSOFA scoring system proposed by Matics was used here to assess the degree of organ injury during the ICU stay.

### Statistical analysis

Categorical variables were expressed as number and percentage, and continuous variables were expressed as median and interquartile range. Normality of continuous data was assessed using the Shapiro-Wilk test. Univariate analysis was done using the Mann-Whitney test or Student’s t-test. For multivariable adjusted analysis, logistic regression modeling was performed. The results were presented as odds ratios as a measure of risk with accompanying95% confidence intervals (C.I.). P values were obtained from the Wald test. Theoptimal cutoff value predicting mortalitywas derived from receiver operating characteristic (ROC) curve analysis by maximizing Youden’s J index. Youden’s J index is defined as J = sensitivity + specificity −1. The point in the ROC curve that maximizes the J value is considered to be optimal for the cutoff point [7]. The statistical analyses were performed using PRISM software (GraphPad Software, San Diego, CA) and Stata 13software (College Station, TX). A two-tailed p< 0.05 was considered statistically significant. Assuming a Type I error rate of 5%, our sample size of 55 survivors and 20 non-survivors in group A resulted in 80% power for detecting a standardized difference (effect size) in d(pSOFA) values of 0.75 (average difference of 3 with standard deviation of 4) between the two groups, based on Student’s t-test. Power analyses were performed using nQuery Advisor version 7.0 (Statistical Solutions Ltd., Cork, Ireland).

## Results

### Demographics and pSOFA values of HSCTsurvivors and non-survivors who were admitted to ICU

**Figure 1:**
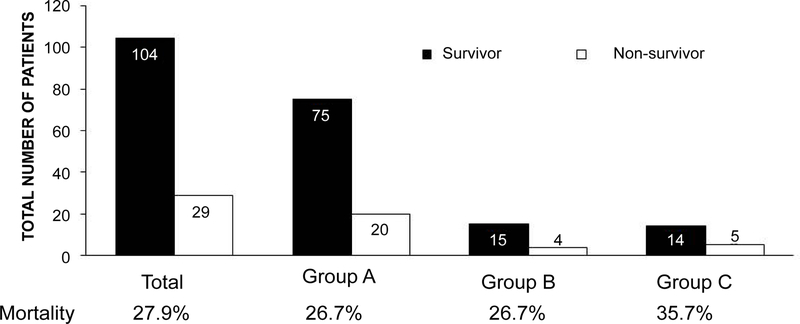
The number of patients who underwent pediatric hematologic stem cell transplantation during the study period in our institution. The number of survivors and non-survivors and the percentages of mortality are shown.

Figure 1 showed the number of survivors or non-survivors who required ICU admission. Survivors was defined as patients who were discharged from the hospital. Overall mortality was 27.9 %. The mortalities of patients in Group A, B and C were 26.7%, 26.7% and 53.7%, respectively. Table 1 showed the characteristics of survivors and non-survivors in Group A, B and C. In Group A, non-survivors were older than survivors. Average and maximum pSOFA values of non-survivors were significantly higher than those of survivors. In addition, the duration of ICU stay for non-survivors was significantly longer than that for survivors. In contrast, there was no statistical difference in age, pSOFA scores and duration of ICU stay between non-survivors and survivors in Group B and C. However, the medianvalues of maximum and average pSOFA scores were higher and the median duration of ICU stay was longer in non-survivors, as in the case for Group A. The sample sizes of Group B and C weresmall, which may explain no statistical significance in these categories between survivors and non-survivors in Group B and C.

We defined injury of each organ system as pSOFA sub score >= 2 as previously described [8]. As expected, the frequency of hematologic injury at the time of ICU admission was higher in Group A and C, both of which represented patients who received HSCT and were admitted to ICU during the same hospital stay (Table 2). In contrast, hematologic injury was seen inonly a half of patients in Group B. Respiratory system was the second most injured organ at the time of admission (Table 2). The ICU admission diagnosis was listed in Table 3. Respiratory distress/ failure was the major cause of ICU admission, which was in line with the data in Table 2.

### Admission pSOFA did not predict mortality but the larger difference between maximum pSOFA and admission pSOFA was associated with mortality

**Figure 2:**
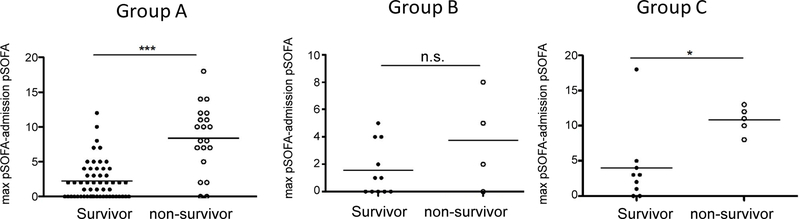
The comparison of difference between maximum pSOFA and admission pSOFA Maximum pSOFA – admission pSOFA was defined as d(pSOFA). d(pSOFA) in Group A, B and C was compared between survivors and non-survivors. Statistical analysis was performed using Mann Whitney test. * and *** denote p< 0.05 and p<0.001, respectively. n.s. = not significant.

Admission pSOFA between survivors and non-survivors did not show any difference in Group A-C, suggesting that this parameter would not serve to predict mortality (Table 1). Here we defined d(pSOFA) as [maximum pSOFA – admission pSOFA]. d(pSOFA) was compared between survivors and non-survivors in Group A-C (Figure 2). d(pSOFA) was statistically larger in non-survivors than in survivors in Group A and C, but not in Group B. Based on this, d(pSOFA) was a better predictor than maximum pSOFA or average pSOFA.

Knowing that d(pSOFA) was larger in non-survivors than in non-survivors in Group A and C, we determined the cut-off value of d(pSOFA) to predict mortality for both groups. The cutoff- value for Group A was equal to and above7 (Table 4). The area under the curve (AUC) was 0.850, which suggested that this cut-off value was a good predictor. The cutoff-value for Group C was equal to and above 8. The AUC was 0.844.

### Type of organ injury associated with mortality

Because pSOFA consists of six different domains, understanding the organ system succumbed toinjury most would be helpful. Because a significant difference in d(pSOFA) between survivors and non-survivors was noted in Group A and C, we examined the progression of each organ injury during ICU stay. We defined the difference in subscore of each organ at the time of admission and maximum pSOFA as d(subscore). d(subscore) was compared for each organ system. In Group A, univariable analysis showed that neurologic, cardiovascular, respiratory, hepatic and renal d(subscore) were significantly higher in non-survivors. Multivariable analysis showed that only neurologic and cardiovascular d(subscore) were significantly higher in non-survivors. We also analyzed Group C similarly. We did not find any statistical significance between survivors and non-survivors. This could be due to a small sample size of Group C.

## Discussion

Here we have shown that 1) pediatric HSCT recipients who required ICU admission demonstrated high mortality, and 2) patients requiring ICU admission after HSCT during the same hospital admission showeda strong correlation between their mortalities and d(pSOFA) scores. Multivariable analysis of Group A showed that neurologic and cardiovascular injuries had worsened significantly morein non-survivors than in survivors.

The pSOFA scores we used in this study was originally reported by Matics et al. [6]. The cardiovascular and renal subscore system was modified to fit for different age group in pediatric population, and respiratory sub-score system incorporatedoxygen saturation (SpO2)/inspired oxygen concentration (FiO_2_) in addition to partial pressure of oxygen (PaO_2_)/FiO_2_. The latter was introduced mainly due to unavailability of PaO_2_/FiO_2_ in a number of pediatric population. Two other pediatric versions of SOFA scoring system were proposed, but neither of them has not been validated yet [9,10]. The majority of HSCT patients are onanti-microbial medications. Thus with the exisitence of organ injury, technically some of them meet the criteria of sepsis. Because SOFA composes of current sepsis diagnosis criteria proposed in Sepsis-3 [11], we chose to test pSOFA here. Because admission pSOFA score was not a good predictor of survival in pediatric HSCT patients, we decided to examine the association between the mortality and the progression of organ injury during ICU stay. d(pSOFA) and d(subscore) could be good parameters to predict outcomes of pediatric HSCT patients. Although respiratory distress/failure was common cause of admission to ICU, worsening neurologic or cardiovascular system was associated with mortality. However, this was a retrospective study in nature and we could not identify clear relationship between respiratory events and cardiovascular/neurological events. Identifying such causative relationship will be of significant help in clinical management.

Limitation of this study is as follows; this is a single center, retrospective study. Validation of utility of these parameters should be done in other institutions, possibly in the form of multi-center studies. Because of retrospective study in nature, there may be potential documentation errors in electronic medical record. Thus, validating this in prospective manner would be a next important step.

In conclusion, we found that d(pSOFA) was strongly associated with mortality in pediatric patients who received HSCT and required ICU admission during the same admission. Subscore analysis showed that neurologic and cardiovascular injuries progressed more in non-survivors than in survivors.

## Figures and Tables

**Table 1:** Characteristics of patients who received HSCT and admitted to ICU Profiles of survivors and non-survivors in Group A, B and C. Age, admission pSOFA, average pSOFA, maximum pSOFA and duration of ICU stay were shown as median [25^th^ percentile, 75^th^ percentile].

Group A				
	**Survivor (n=55)**	**Non-survivor (n=20)**	**P value**	**Odds ratio (95% C.I.)**
Age (years)	3.75 [1.00, 9.17]	12.25 [2.48, 16.27]	0.029*	1.09 [1.01–1.17]
Male Gender	34 (61.8%)	9 (45.0%)	0.196	0.51 [0.18–1.42]
Admission pSOFA	7.00 [5.00, 9.00]	8.50 [6.00, 11.25]	0.232	1.08 [0.95–1.23]
Average pSOFA	6.00 [5.19, 7.85]	12.70 [10.14, 15.67]	< 0.001*	2.23 [1.49–3.33]
Maximum pSOFA	9.00 [7.00, 13.00]	18.00 [15.75, 19.25]	< 0.001*	1.67 [1.32–2.11]
Duration of ICU stay	11.00 [5.00, 32.00]	33.50 [11.00, 51.50]	0.446	1.01 [0.99–1.02]
Group B				
	**Survivor (n=11)**	**Non-survivor (n=4)**	**P value**	**Odds ratio (95% C.I.)**
Age (years)	7.33 [2.25, 9.75]	14.58 [13.46, 15.27]	0.113	1.24 [0.95, 1.62]
Male Gender	5 (45.5%)	0 (0%)	n/a	n/a
Admission pSOFA	5.00 [2.50, 7.00]	7.25 [5.00, 9.25]	0.541	1.08 [0.84, 1.39]
Average pSOFA	3.75 [2.89, 5.83]	8.60 [7.85, 9.12]	0.074	1.78 [0.95. 3.35]
Maximum pSOFA	5.00 [4.00, 11.00]	11.00 [10.00, 12.75]	0.222	1.17 [0.91, 1.50]
Duration of ICU stay	4.00 [1.00, 5.00]	4.00 [4.00, 31.50]	0.307	1.04 [0.97, 1.11]
Group C				
	**Survivor (n=9)**	**Non-survivor (n=5)**	**P value**	**Odds ratio (95% C.I.)**
Age (years)	6.42 [2.58, 13.42]	5.50 [2.42, 8.92]	0.428	0.92 [0.74, 1.33]
Male Gender	7 (77.8%)	4 (80.0%)	0.923	1.14 [0.08, 16.95]
Admission pSOFA	6.00 [4.00, 7.00]	6.00 [5.00, 9.00]	0.816	0.96 [0.68, 1.35]
Average pSOFA	6.21 [5.33, 7.75]	12.31 [8.69, 14.83]	0.111	1.29 [0.99, 1.68]
Maximum pSOFA	10.00 [6.00, 11.00]	18.00 [18.00, 19.00]	0.064	1.29 [0.99, 1.68]
Duration of ICU stay	9.00 [7.00, 11.00]	29.00 [21.00, 63.00]	0.167	1.03 [0.99, 1.07]

* denotes statistical significance.

C.I., confidence interval; n/a, not available.

**Table 2:** Type of organ injury at the time of ICU admission The frequency of each organ injury at the time of ICU was shown. Each organ injury was defined by pSOFAsubscore>= 2.

Group A						
	**Neurologic**	**Cardiovascular**	**Respiratory**	**Hepatic**	**Renal**	**Hematologic**
Survivor	23.6%	21.8%	45.5%	20.0%	16.4%	92.7%
Non-survivor	20.0%	35.0%	65.0%	30.0%	20.0%	95.0%
Group B						
	**Neurologic**	**Cardiovascular**	**Respiratory**	**Hepatic**	**Renal**	**Hematologic**
Survivor	18.2%	27.3%	36.4%	9.1%	18.2%	54.5%
Non-survivor	50.0%	25.0%	75.0%	50.0%	0%	50.0%
Group C						
	**Neurologic**	**Cardiovascular**	**Respiratory**	**Hepatic**	**Renal**	**Hematologic**
Survivor	22.2%	0%	22.2%	33.3%	0%	88.9%
Non-survivor	0%	0%	40.0%	20.0%	20.0%	80.0%

**Table 3:** Admission diagnosis to ICU Causes of admission to ICU were shown for each group. Number and percentage of patients are shown.

Group A	
Respiratory distress/ failure	60 (80.0%)
Hemodynamic instability/Septic shock	9 (12.0%)
Others	6 (8.0%)
Group B	
Respiratory distress/ failure	12 (80.0%)
Hemodynamic instability/ Septic shock	2 (13.3%)
Others	1 (6.7%)
Group C	
Respiratory distress/ failure	12 (85.8%)
Hemodynamic instability/ Septic shock	1 (7.1%)
Others	1 (7.1%)

**Table 4:** The cutoff value of d(pSOFA) to predict mortality d(pSOFA) was defined as [maximum pSOFA – admission pSOFA]. The cutoff value of d(pSOFA) to best predict mortality was obtained from Youden-J index. C.I., confidence interval; AUC, area under the curve.

Group	d(pSOFA) cut-off value	AUC [95% C.I.]
Group A	=< 7	0.850 [0.733– 0.966]
Group C	=< 8	0.844 [0.684–0.972]

**Table 5:** Correlation between type of organ injury and mortality Correlation between progression of organ injury and mortality was examined using univariable and multivariable analyses for Group A and C. d(subscore), defined as in the text, was used for this purpose and shown as median [25th percentile, 75th percentile] (mean).

Group A Univariateanalysis				
Type of organ injury	Survivor (n=55)	Non-survivor (n=20)	P value	Odds ratio (95% C.I.)
Neurological injury	0.00 [0.00, 1.00](0.64)	4.00 [3.00, 4.00](3.00)	< 0.001*	2.92 [1.86–4.58]
Cardiovascular injury	0.00 [0.00, 1.00](0.18)	1.50[0.00, 3.00](1.60)	< 0.001*	2.96 [1.66–5.26]
Respiratory injury	0.00 [0.00,0.00](0.65)	1.50[0.25, 2.75](1.55)	0.010*	1.74 [1.14–2.65]
Hepatic injury	0.00 [0.00, 0.00](0.31)	2.00[0.00, 2.00](1.20)	0.003*	2.34 [1.34–4.11]
Renal injury	0.00 [0.00, 1.00](0.32)	1.00[0.00,2.00](1.00)	0.019*	1.86 [1.11–3.12]
Hematologic injury	0.00 [0.00,0.00](0.09)	0.00[−1.00, 1.00](0.05)	0.847	0.94 [0.50–1.77]
Multivariable analysis				
Type of organ injury		P value	Odds ratio (95% C.I.)	
Neurological injury		0.003*	2.27 [1.32–3.93]	
Cardiovascular injury		0.015*	2.69 [1.21–5.99]	
Respiratory injury		0.117	0.57 [0.28–1.15]	
Hepatic injury		0.120	1.90 [0.85–4.29]	
Renal injury		0.122	2.01 [0.83–4.85]	
Group C Univariate analysis				
Type of organ injury	Survivor (n=9)	Non-survivor (n=5)	P value	Odds ratio (95% C.I.)
Neurological injury	0.00[0.00,1.00](0.44)	3.00[2.50, 4.00](3.20)	n/a	n/a
Cardiovascular injury	0.00[0.00,2.00](0.78)	3.00[1.00, 3.50](2.40)	0.100	1.98 [0.88–4.48]
Respiratory injury	0.00[0.00,2.00](0.89)	3.00[2.00, 4.00](3.00)	0.065	5.46 [0.90–33.18]
Hepatic injury	0.00[0.00,1.50](0.78)	1.00[0.00,2.00](1.00)	0.738	1.17 [0.47–2.88]
Renal injury	0.00[0.00, 0.50](0.11)	0.00[0.00,2.00](0.80)	0.235	2.63 [0.53–12.92]
Hematologic injury	1.00[0.00,1.00](1.00)	0.00[−0.50,1.50](0.40)	0.416	0.64 [0.21–1.89]

* denotes statistical significance.

C.I., confidence interval.
